# Depression-Related Mechanistic and Translational Evidence for *Centella asiatica* and Its Triterpenoids: A Scoping Review

**DOI:** 10.3390/life16081345

**Published:** 2026-08-16

**Authors:** Pimol Kanchalearnpong, Auemphon Mordmuang, Lavanya Goodla, Weeratian Tawanwongsri

**Affiliations:** 1 Division of Child and Adolescent Psychiatry, Department of Psychiatry, School of Medicine, Walailak University, Nakhon Si Thammarat 80160, Thailand; 2 Department of Medical Science, School of Medicine, Walailak University, Nakhon Si Thammarat 80160, Thailand; 3 Department of Biochemistry and Molecular Biology, School of Medicine, University of New Mexico, Albuquerque, NM 87131, USA; 4 Division of Dermatology, Department of Internal Medicine, School of Medicine, Walailak University, Nakhon Si Thammarat 80160, Thailand

**Keywords:** *Centella*, depression, triterpenes, phytotherapy, disease models, animal, brain-derived neurotrophic factor, oxidative stress, neuroinflammation

## Abstract

Background: Depression is a multifactorial psychiatric disorder involving monoaminergic, neurotrophic, inflammatory, oxidative, and stress-response pathways. *Centella asiatica* and its triterpenoids have demonstrated neuroprotective and stress-modulating properties, but depression-focused evidence remains fragmented. Objective: The aim of this scoping review was to map the available evidence on *C. asiatica*, its standardized extracts, and its bioactive triterpenoids in relation to depression-related outcomes and mechanisms. Methods: A protocol was registered before formal screening, full-text assessment, data charting, and evidence synthesis (INPLASY202670003). Scopus, PubMed/MEDLINE, the Cochrane Library, and ClinicalTrials.gov were searched from inception to 2 July 2026. Eligible studies included human, animal, cell-based, and ex vivo studies reporting depression, depressive-like behavior, antidepressant-like effects, or mechanisms explicitly linked to depression-related pathophysiology. Two reviewers independently screened the identified records and charted the data using a standardized form, with disagreements resolved through consensus or consultation with a third reviewer. The findings were synthesized descriptively and narratively. Results: A total of 14 studies published between 2008 and 2025 were included, comprising 13 preclinical studies and 1 open-label human study. No eligible cell-based or ex vivo studies were identified. The included studies primarily examined *C. asiatica* extracts and isolated triterpenoids, particularly asiaticoside and asiatic acid. Most studies reported favorable depression-related or antidepressant-like findings. The principal reported mechanisms involved BDNF/CREB-related signaling and neuroplasticity, monoaminergic regulation, attenuation of neuroinflammation and oxidative stress, and modulation of the hypothalamic–pituitary–adrenal axis. However, the evidence base is heterogeneous and predominantly derived from animal models. Conclusions: The available evidence supports the biological plausibility and preclinical antidepressant-like activity of *C. asiatica* and its triterpenoids; however, the evidence remains insufficient to establish clinical efficacy in humans. Future studies should prioritize standardized preparations, dose justification, pharmacokinetic and safety evaluation, validated depression-specific outcomes, and rigorously controlled clinical trials.

## 1. Introduction

Depression is one of the most common psychiatric disorders and a major public health problem, with the number of incident cases worldwide increasing from approximately 172 million in 1990 to 258 million in 2017 [1]. Although the monoamine deficiency hypothesis has long shaped its treatment, this framework does not fully account for the pathophysiology of the disorder, which is increasingly understood to be multifactorial and network-based rather than a simple chemical imbalance [2,3]. The contributing mechanisms include dysregulation of the hypothalamic–pituitary–adrenal (HPA) axis, neuroinflammation, oxidative stress, mitochondrial dysfunction, impaired brain-derived neurotrophic factor (BDNF) signaling, and reduced neuroplasticity [2,4]. Standard management combines pharmacotherapy, such as antidepressants, with psychotherapy and other interventions in a stepped-care approach. However, some patients experience an inadequate response or adverse effects that may require modification of the pharmacotherapy under the supervision of a qualified healthcare professional [4,5]. Although complementary and traditional medicines are used in many settings [4,6], medicinal plants and plant-derived compounds remain investigational in the context of depression because their clinical efficacy and safety have not been adequately established. They should not be considered substitutes for evidence-based treatment, but their potential effects on multiple depression-related pathways warrant further preclinical and clinical investigation [4,7].

*Centella asiatica* (L.) Urban, commonly known as gotu kola or Indian pennywort, is a traditional medicinal plant widely used across Asia. It has been used for centuries in Ayurvedic, Unani, and traditional Chinese medicine as a rejuvenative tonic for cognitive and mental disorders [8,9]. Its principal bioactive constituents are pentacyclic triterpenoids, including asiatic acid, asiaticoside, madecassic acid, madecassoside, and madasiatic acid [9]. Among these compounds, asiaticoside, madecassoside, and asiatic acid have demonstrated blood–brain barrier permeability in vitro, supporting their potential to act on central nervous system targets [10]. These compounds have been studied for their neuroprotective, antioxidant, anti-inflammatory, neurotrophic, and stress-modulating effects [11,12], which overlap mechanistically with several pathways implicated in the pathophysiology of depression. Consistent with this biological plausibility, preclinical studies have suggested antidepressant or depression-like effects. In a chronic unpredictable mild stress model, asiaticoside improved depressive-like behaviors, as demonstrated by reduced immobility in the forced swim and tail suspension tests and restored sucrose preference. These behavioral effects were accompanied by increased hippocampal serotonin and norepinephrine levels, reduced NF-κB/NLRP3-related neuroinflammatory signaling, and activation of the PKA/pCREB/BDNF pathway [13]. Complementary evidence indicates that *C. asiatica* increases BDNF expression in the prefrontal cortex [14], enhances Nrf2-mediated antioxidant responses and reduces oxidative stress [15], improves mitochondrial function and neurotrophic signaling [16], and promotes neuronal differentiation, axodendritic maturation, and synaptogenesis [17]. Together, these findings suggest several plausible mechanisms, including monoaminergic regulation, BDNF/CREB signaling, stress-response modulation, and attenuation of neuroinflammation and oxidative stress. These mechanisms provide a rationale for systematically mapping the available depression-related evidence on *C. asiatica* and its triterpenoids.

Despite this mechanistic rationale, depression-related evidence on *C. asiatica* remains scattered across heterogeneous preclinical and clinical studies, with animal models predominating and human evidence remaining limited [8,11]. These studies differed substantially in the preparations or isolated compounds evaluated, doses, routes of administration, treatment durations, experimental models, comparators, and outcome measures [8,12,13]. Furthermore, some studies directly assessed depressive-like behavior, whereas others evaluated mechanistic outcomes, such as BDNF/CREB signaling, monoaminergic regulation, and neuroinflammation, that have been implicated in depression-related pathophysiology [13,14]. Important translational gaps persist, particularly in the scarcity of clinical evidence, poor triterpenoid bioavailability, and unresolved questions regarding extract standardization, dosing, safety, and mechanistic validation [4,11,16]. To the best of our knowledge, this body of evidence has not yet been clearly mapped in a depression-focused manner, and it remains unclear which compounds, models, outcomes, and mechanisms are the most studied. Therefore, a scoping review is well-suited for mapping the available literature and identifying research gaps.

The scarcity of human evidence underscores the need to systematically map the available preclinical and clinical literature. Accordingly, this scoping review maps the evidence on *C. asiatica*, its standardized extracts, and its bioactive triterpenoids in relation to depression, depressive-like behavior, antidepressant-like effects, and biological mechanisms explicitly linked to depression-related pathophysiology. The review aims to identify the compounds, experimental models, outcomes, and mechanisms most frequently studied and to define key translational gaps, including those related to clinical evidence, dosing, extract characterization, outcome measurement, safety, and mechanistic validation. The review was guided by the following research question: What human, animal, cell-based, or ex vivo evidence is available on the effects of *C. asiatica*, its standardized extracts, and its bioactive triterpenoids on depression, depressive-like behavior, antidepressant-like effects, and depression-linked mechanisms?

## 2. Materials and Methods

### 2.1. Protocol and Registration

This scoping review was conducted in accordance with the JBI methodology for scoping reviews [18] and is reported in accordance with the Preferred Reporting Items for Systematic Reviews and Meta-Analyses extension for Scoping Reviews (PRISMA-ScR) [19]. The protocol was defined a priori and registered with the International Platform of Registered Systematic Review and Meta-Analysis Protocols (INPLASY) on 2 July 2026 (registration number INPLASY202670003; https://doi.org/10.37766/inplasy2026.7.0003). The preliminary searches and protocol registration occurred on the same calendar date. At the time of registration, formal title/abstract screening, full-text assessment, data charting, and evidence synthesis had not begun. The protocol was subsequently amended on 5 July 2026, before final screening, data extraction, and evidence synthesis, to clarify and narrow the depression-focused eligibility criteria. The amendment did not change the search strategy or the retrieved record set; rather, the same records were re-screened using the amended criteria, and the final study selection was based on these criteria. The complete PRISMA-ScR checklist is provided in Supplementary File S1.

### 2.2. Eligibility Criteria

The eligibility criteria were structured using the Population, Concept, and Context (PCC) framework recommended by the JBI for scoping reviews, focusing on depression and depression-related conditions. Eligible sources included human, animal, cell-based, and ex vivo studies on *C. asiatica* (L.) Urb. or its derived preparations and constituents, including gotu kola, standardized *C. asiatica* extracts, total triterpenes, asiatic acid, asiaticoside, madecassic acid, madecassoside, madasiatic acid, and other clearly identified *C. asiatica*-derived compounds, in relation to depression, depressive-like behavior, antidepressant-like effects, or mechanisms explicitly linked to depression-related pathophysiology. Studies examining polyherbal formulations were excluded unless they contained a *C. asiatica*-only comparison arm, standardized the formulations to *C. asiatica* triterpenoids, or reported extract-specific findings attributable to *C. asiatica*. Studies were eligible if they reported at least one depression-related outcome—depression, depressive symptoms, depressive-like behavior, antidepressant-like effects, major depressive disorder, or scores on a validated depression rating scale—or employed an established depression-related animal model or behavioral assay, including chronic unpredictable mild stress, chronic mild stress, chronic restraint stress as a depression model, olfactory bulbectomy, the forced swim test, the tail suspension test, or the sucrose preference test. Mechanistic studies were eligible when the biological outcomes assessed—such as HPA axis activity, cortisol or corticosterone, monoamine neurotransmission (serotonin, dopamine, and norepinephrine/noradrenaline), BDNF/CREB signaling, neuroinflammation and NF-κB/NLRP3 signaling, oxidative stress and Nrf2 signaling, mitochondrial function, neuroplasticity, neurogenesis, or synaptic outcomes—were explicitly linked by the authors to depression or depressive-like behavior. Human studies were eligible whether they enrolled healthy participants or clinical populations and reported a depression-related outcome.

Studies focused solely on anxiety, stress without depression-related interpretation, or mood outcomes without depression relevance were excluded, as were studies focused only on cognition or on non-depressive neurological conditions such as Alzheimer’s disease, Parkinson’s disease, stroke, epilepsy, traumatic brain injury, or general neuroprotection. Studies were eligible only if they additionally reported depression-related outcomes or provided mechanistic evidence explicitly interpreted in relation to depression or depression-like behavior. Eligible study designs included human randomized controlled trials, non-randomized clinical studies, observational human studies, animal experimental studies, and cell-based or ex vivo mechanistic studies that reported original data. Reviews, editorials, commentaries, letters, book chapters, protocols, and conference abstracts lacking sufficient original data, as well as pure phytochemical or extraction studies without depression-related outcomes, were excluded. Computational studies, including molecular docking, molecular dynamics simulations, network pharmacology, and in silico absorption, distribution, metabolism, excretion, and toxicity (ADMET) analyses, were excluded unless they were reported in an article that also presented eligible cell-based, animal, or human experimental data. Eligibility was restricted to full-text articles published in English, a pragmatic constraint acknowledged as a potential source of language bias in the Limitations section.

### 2.3. Information Sources

Four electronic databases were searched from inception to 2 July 2026, without a lower date limit: Scopus, PubMed/MEDLINE, the Cochrane Library, and ClinicalTrials.gov. The database searches were completed on 2 July 2026, the same calendar date on which the protocol was registered, before formal title/abstract screening, full-text assessment, data charting, and evidence synthesis began. Scopus and PubMed/MEDLINE served as the primary bibliographic databases for published peer-reviewed literature, providing broad multidisciplinary and biomedical coverage across cell-based, animal, and human studies. Within the Cochrane Library, the search focused on the Cochrane Central Register of Controlled Trials (CENTRAL) to identify reports of randomized and quasi-randomized clinical trials. ClinicalTrials.gov was searched as a trial registry to identify registered clinical trials, whether ongoing, completed, unpublished, or terminated, and to locate any corresponding full-text publications. Registry records without an eligible full-text article were not treated as included sources of evidence but were used to characterize the extent of registered clinical activity, consistent with the review’s secondary objective of mapping translational and clinical gaps. To supplement the electronic searches, the reference lists of all included studies and relevant reviews were manually screened to identify additional eligible sources. To characterize current clinical development, the status of potentially relevant registry records was rechecked on 11 July 2026. Ongoing studies were defined as those listed as recruiting, not yet recruiting, active but not recruiting, or enrolling by invitation.

### 2.4. Search Strategy

The search strategy combined three conceptual blocks using Boolean logic: (i) terms for *C. asiatica* and its preparations and bioactive constituents (e.g., “*Centella asiatica*,” “gotu kola,” “*Hydrocotyle asiatica*,” “Indian pennywort,” standardized extracts, total triterpenes and total triterpenic fraction, asiatic acid, asiaticoside, madecassic acid, madecassoside, and madasiatic acid); (ii) terms for depression-related and mechanistic outcomes (e.g., depression, depressive- or depression-like behavior, antidepressant-like effects, major depressive disorder, mood, anxiety, and stress, including validated behavioral paradigms such as chronic unpredictable mild stress, forced swim, tail suspension, and sucrose preference, as well as HPA axis markers, monoamines, BDNF/CREB, neuroinflammation, oxidative stress, Nrf2, mitochondrial function, and neuroplasticity); and (iii) terms anchoring the neuropsychiatric or central nervous system context (e.g., brain, hippocampus, cortex, neuron, astrocyte, microglia, and behavioral or emotional descriptors). Within each block, synonyms and lexical variants were combined using the OR operator, and the three blocks were combined using AND. Phrase searching, truncation, and proximity operators were used to capture spelling and terminological variants. The search strategy was first constructed in Scopus and then translated and adapted to the syntax and field structure of each remaining database. Limits were applied to the English language and publication year through 2026, with no lower date limit. This strategy was intentionally broad and sensitive, encompassing anxiety-, stress-, and mood-related terms in addition to depression-specific terms. The depression-focused eligibility criteria were applied during study selection rather than through additional search restrictions, thus preserving sensitivity while achieving the intended depression focus at the screening stage. The complete search strategies for all databases, along with the number of records retrieved from each, are provided in Supplementary Table S1.

### 2.5. Study Selection

All records retrieved from the electronic searches were imported into EndNote X9 (Clarivate, Philadelphia, PA, USA), and duplicate records were removed prior to screening. Study selection proceeded in two stages. First, two reviewers independently screened the titles and abstracts of all remaining records in EndNote against the predefined eligibility criteria, and records judged as clearly irrelevant by both reviewers were excluded. Second, the full texts of all potentially eligible records were retrieved and independently assessed by two reviewers using the same criteria. Reasons for exclusion at the full-text stage were recorded and included the wrong intervention or compound; the absence of any depression-related outcome and mechanistic evidence explicitly linked to depression or depressive-like behavior; a focus restricted to anxiety, stress without depression-related interpretation, mood without depression relevance, cognition, or a non-depression neurological condition; an ineligible study design; a review or non-empirical publication without original data; a pure phytochemical or extraction study without depression-related outcomes; a purely computational study without eligible experimental data; and full text unavailable in English. Disagreements at either stage were resolved through discussion, and those that could not be reconciled were referred to a third reviewer.

### 2.6. Data Extraction and Charting

Data were charted using a standardized form developed a priori by the review team in accordance with the JBI approach to data extraction for scoping reviews. The form was piloted on a small sample of included studies before full charting to confirm that it captured all relevant variables and to promote consistency among reviewers. Any modifications made to the form during the review were documented, in keeping with the iterative nature of charting in scoping reviews. Two reviewers independently charted the data. Discrepancies were resolved through discussion, and any remaining unresolved data were referred to a third reviewer. Data are presented as reported in the primary sources. The variables included bibliographic details (first author, year of publication, and country); study design; model or population (human, animal, cell-based, or ex vivo); the intervention or exposure, including the specific *C. asiatica* preparation or constituent, comparator, dose, route of administration, and treatment duration; the depression-related outcomes and mechanistic endpoints assessed; reported safety findings; and the authors’ key conclusions. To support the planned synthesis, each source was further categorized by intervention or preparation type (crude extract, standardized extract, total triterpenes, or isolated compound), depression-related outcome type, and depression-linked biological pathway(s), as defined in the eligibility criteria (Section 2.2). For descriptive summary statistics, intervention categories were mutually exclusive: extract only, isolated triterpenoid only, or both extract and isolated triterpenoid. Studies evaluating both intervention types were assigned to the combined category to avoid double counting.

### 2.7. Data Analysis and Presentation

The charted data were summarized using a combined numerical and narrative approach. No meta-analysis was undertaken, and a formal quantitative synthesis was considered inappropriate given the anticipated heterogeneity across the study models, *C. asiatica* preparations, outcome measures, and mechanistic endpoints. Study characteristics were summarized descriptively using counts and frequencies, such as the distribution of study designs, experimental models, preparations, and outcome domains, and presented in a tabular form alongside a narrative account of the findings. The included studies were grouped by study type (cell-based or ex vivo, animal, and human studies), intervention or preparation type (crude extracts, standardized extracts, total triterpenes, and isolated compounds), depression-related outcome type, and depression-linked mechanistic pathways examined, including those related to HPA axis activity, monoamine neurotransmission, BDNF/CREB signaling, neuroinflammation, oxidative stress, Nrf2 signaling, mitochondrial function, and neuroplasticity. For descriptive summaries, chronic mild stress (CMS), chronic unpredictable mild stress (CUMS), chronic unpredictable stress (CUS), and unpredictable chronic stress (UCS) were grouped under the umbrella category of chronic unpredictable stress paradigms. The terminology reported by each original study was retained in the tables. Building on this mapping, and in line with the secondary objective of the review, translational gaps were identified with respect to clinical evidence, dosing, extract standardization, outcome measurement, safety, and mechanistic validation.

Formal critical appraisal was not conducted because the purpose of this scoping review was to map the extent, characteristics, and gaps in the evidence rather than to estimate intervention effectiveness. However, the key methodological and reporting limitations of the included studies, including study design, model type, extract standardization, mechanistic validation, and safety reporting, were described and incorporated into the narrative synthesis.

## 3. Results

**Figure 1:**
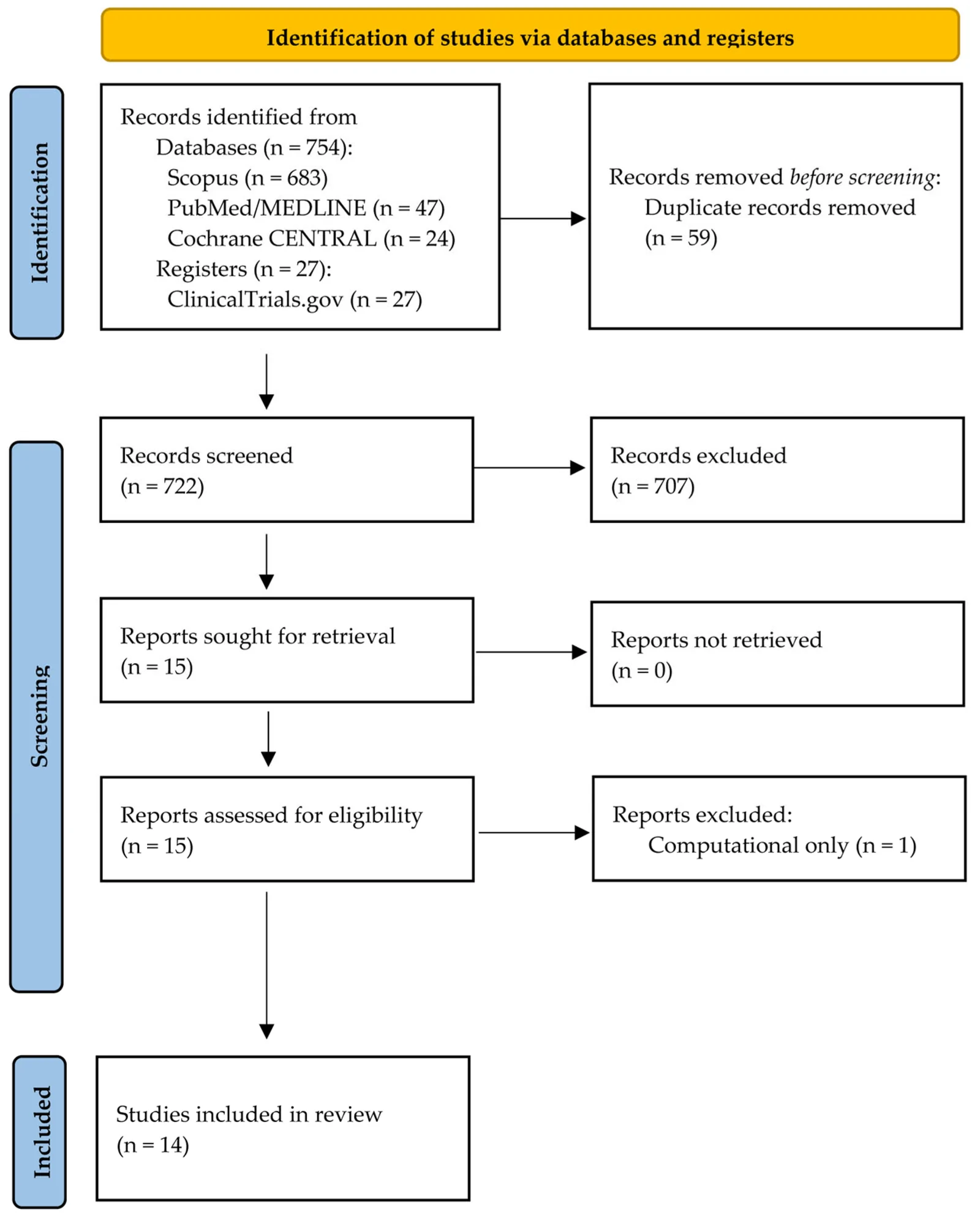
PRISMA 2020 flow diagram showing the identification, screening, eligibility assessment, and inclusion of studies in the scoping review. Abbreviations: CENTRAL, Cochrane Central Register of Controlled Trials; MEDLINE, Medical Literature Analysis and Retrieval System Online; PRISMA, Preferred Reporting Items for Systematic Reviews and Meta-Analyses.

The database and registry searches identified 781 records, including 754 from the three databases and 27 from ClinicalTrials.gov. After removing 59 duplicates, 722 records were screened by title and abstract, and 707 were excluded at this stage. Fifteen full-text reports were assessed for eligibility, and one was excluded because it was a computational study that did not include eligible experimental data. Fourteen studies were included in the final analysis. The study selection process is illustrated in Figure 1.

Among the 27 ClinicalTrials.gov records, five studies were listed as ongoing or active. None of these studies enrolled participants with depression or evaluated antidepressant efficacy. The only CNS-related study was NCT05591027, which evaluated oral *C. asiatica* in individuals with mild cognitive impairment or Alzheimer’s disease. The remaining studies investigated a Brahmi–gotu kola oil massage intervention for sleep disturbances (NCT07274371), oral asiaticoside for overweight or obesity (NCT07241533), oral madecassoside for chronic graft-versus-host disease (NCT07606703), and intracanal asiaticoside for apical periodontitis (NCT06566508).

Most studies were conducted in Asia, predominantly in China (*n* = 6) and India (*n* = 4), with additional studies conducted in Malaysia (*n* = 2), Brazil (*n* = 1), and Poland (*n* = 1). Preclinical evidence was derived chiefly from rodent models, including mice (*n* = 7) and rats (*n* = 5), along with one study that combined an adult zebrafish model with in silico molecular docking [20]. Chronic unpredictable stress paradigms were used in eight studies (57.1%), comprising CMS in two studies, CUMS in four, CUS in one, and UCS in one zebrafish study [20]. Other models included olfactory bulbectomy, chronic restraint stress, maternal deprivation, and acute forced swim or tail suspension tests in non-stressed animals. Two studies used acute behavioral despair tests in non-stressed animals. The animals were predominantly male, and the reported group sizes ranged from 6 to 12. The single human study was a 60-day open-label clinical study of 33 adults (18 men and 15 women) with generalized anxiety disorder accompanied by depressive and stress symptoms [21].

Four studies (28.6%) evaluated *C. asiatica* extracts only, nine (64.3%) evaluated isolated triterpenoids only, and one (7.1%) evaluated both an extract and an isolated triterpenoid. Asiaticoside (*n* = 7) and asiatic acid (*n* = 3) were the most frequently studied compounds, while madecassoside and madecassic acid were examined less often. Oral administration was the most common route, and treatment durations generally ranged from 14 to 60 days. Some studies used acute dosing, whereas others employed intranasal [22] or intraperitoneal administration [20,23]. Comparators included fluoxetine, escitalopram, imipramine, and other reference antidepressants. The mapped outcomes centered on depressive-like or antidepressant-like behavior, assessed alongside depression-related mechanisms—most commonly monoaminergic regulation, BDNF/CREB signaling, neuroinflammation, hypothalamic–pituitary–adrenal axis activity, and oxidative stress—with less frequent examination of neuroplasticity, mitochondrial function, gut microbiota [24], and ferroptosis [25]. None of the included studies reported primary safety, adverse event, toxicity, or tolerability data from their own cohorts; therefore, no conclusions regarding safety or tolerability could be drawn from the included evidence.

In summary, the included studies varied substantially in design, experimental model, intervention type, dose, route of administration, treatment duration, and comparator. Most studies were preclinical and used rodent models of depression-like behavior or antidepressant-like activity, whereas human evidence was limited to one clinical study. These interventions included *C. asiatica* extracts, standardized preparations, and isolated triterpenoids, with asiaticoside and asiatic acid being the most frequently investigated compounds. Treatment approaches also differed across studies, ranging from acute administration to several weeks of repeated dosing. Details of the intervention characteristics and experimental models are presented in Table 1.

Depression-related outcomes were assessed mainly through preclinical behavioral assays, with most studies using the sucrose preference, forced swim, tail suspension, open field, and related stress behavior tests. Most studies reported favorable depression-related or antidepressant-like effects, although one study reported mixed findings when asiaticoside was used alone or in combination with borneol. Human evidence was limited to one study that reported changes in depression-related scores among participants with generalized anxiety disorder. Details of depression-related outcomes and the main findings are presented in Table 2.

The mechanistic outcomes were heterogeneous but clustered around several depression-linked pathways, particularly monoaminergic signaling, BDNF-related neurotrophic pathways, HPA axis activity, neuroinflammation, and oxidative stress. Few studies examined mitochondrial or synaptic integrity, gut microbiota, ferroptosis, or transporter-binding mechanisms. Safety reporting was limited across the included studies, and most translational gaps were related to the lack of confirmation in humans, limited safety data, single-model designs, incomplete extract characterization, or insufficient mechanistic validation. Details of the depression-linked mechanisms, safety findings, and translational gaps are presented in Table 3.

## 4. Discussion

### 4.1. Main Findings and Interpretation Across Non-Human and Human Evidence

This scoping review identified 14 studies published between 2008 and 2025 that examined the effects of *C. asiatica* or its triterpenoids on depression and depression-related mechanisms. The evidence base is overwhelmingly preclinical, comprising 13 non-human studies and only a single human clinical study [21]. Most of these studies originated in Asia, particularly China and India. Rodents constituted the predominant experimental model, with one study employing an adult zebrafish paradigm supported by in silico molecular docking [20]. Chronic unpredictable stress paradigms and their reported variants (CMS, CUMS, CUS, and UCS) were the most frequently used models. Other models included olfactory bulbectomy, chronic restraint stress, maternal deprivation, and acute forced swim and tail suspension tests in non-stressed animals. Isolated triterpenoids, chiefly asiaticoside and asiatic acid, were investigated more often than whole or standardized extracts. These intervention types should be interpreted separately because whole or standardized *C. asiatica* extracts are multicomponent preparations, whereas isolated triterpenoids represent individual compounds with distinct pharmacological properties. Accordingly, findings for isolated asiaticoside, asiatic acid, or other triterpenoids should not be extrapolated to whole or standardized extracts, and vice versa. Similarities in reported antidepressant-like outcomes or mechanistic pathways do not establish pharmacological equivalence between these intervention types. Most studies reported improvements in depression-related or antidepressant-like outcomes. However, the apparent consistency of these favorable findings should not be interpreted as high-certainty evidence. The preclinical studies generally involved small sample sizes, predominantly male animals, single experimental models, and limited safety reporting, and no formal risk-of-bias or certainty-of-evidence assessment was performed. The reported depression-linked mechanisms clustered into five principal domains: (1) BDNF/CREB-related signaling and neuroplasticity [13,22,24,25,28,29,31]; (2) monoaminergic regulation [13,20,24,29,30]; (3) attenuation of neuroinflammation [13,24,25,29,32]; (4) reduction in oxidative stress [23,25,31,32]; and (5) modulation of the hypothalamic–pituitary–adrenal axis [20,22,24]. Less frequently investigated mechanisms included mitochondrial function, gut microbiota modulation, and ferroptosis. Most mechanistic findings were associative, based on concurrent changes in behavioral outcomes and biomarkers following treatment. More direct experimental support was limited to studies using pharmacological manipulation: TrkB inhibition with K252a blocked the antidepressant-like effects of asiaticoside [28], while serotonergic and noradrenergic receptor antagonists reduced the antidepressant-like effects of asiatic acid [30]. These findings provide stronger support for the involvement of BDNF/TrkB and monoaminergic pathways than biomarker associations alone; however, they do not establish that these mechanisms are universally causal across *C. asiatica* extracts, isolated triterpenoids, or experimental models. Other reported pathways should therefore be interpreted primarily as proposed or associative mechanisms.

**Figure 2:**
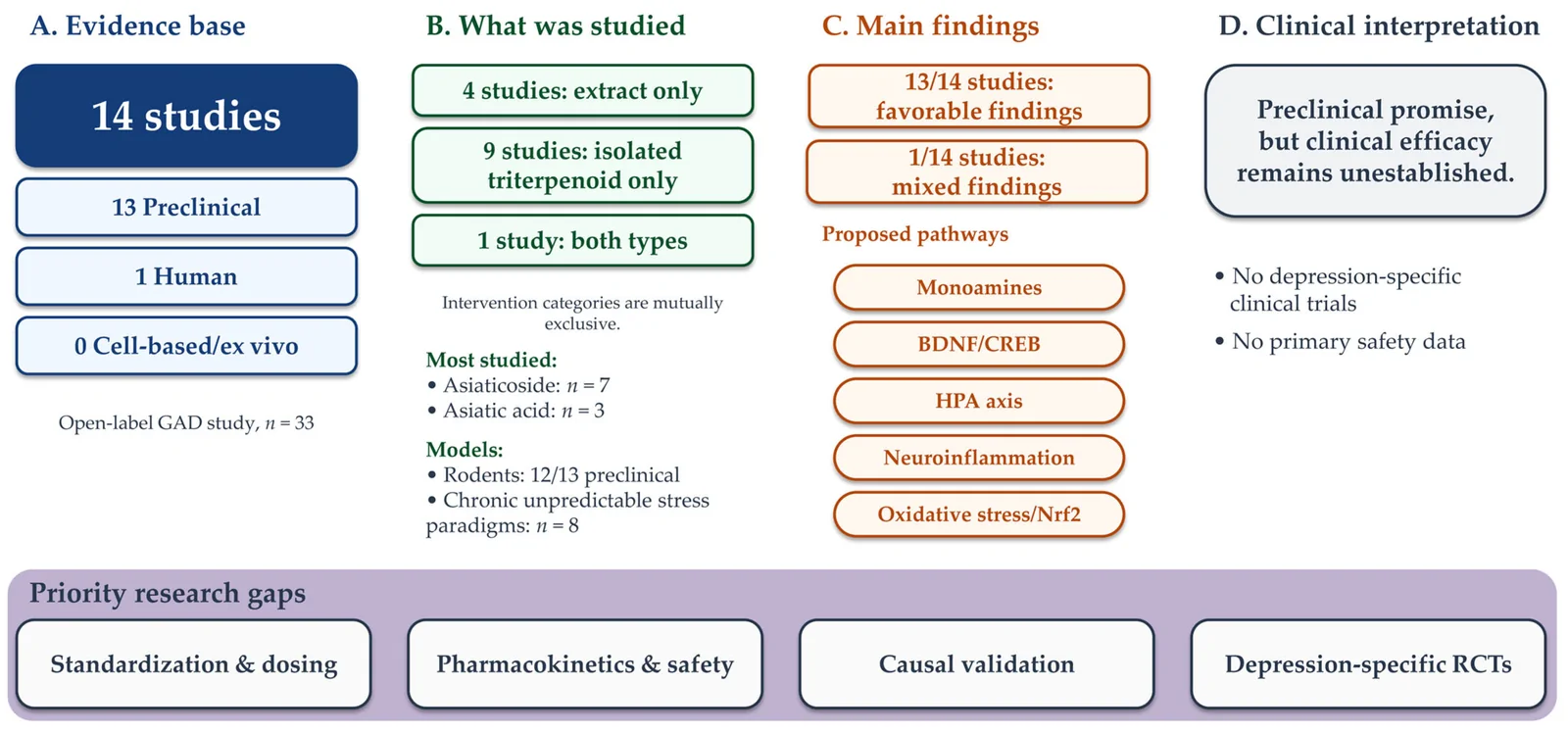
An evidence map and translational interpretation of *Centella asiatica* and its triterpenoids in depression-related research: (**A**) distribution of the reviewed evidence; (**B**) interventions and experimental models; (**C**) depression-related findings and reported or proposed biological pathways; and (**D**) clinical interpretation and priority research gaps. In panel B, intervention categories are mutually exclusive; the category “both types” indicates studies evaluating both a *C. asiatica* extract and an isolated triterpenoid. The pathways represent associations or mechanisms reported or proposed in the included studies and should not be interpreted as established causal mechanisms or evidence of clinical efficacy. The predominance of favorable findings is descriptive and should not be interpreted as evidence of high certainty. Unless otherwise specified, *n* denotes the number of studies; for the open-label GAD study, *n* = 33 denotes the number of participants. BDNF, brain-derived neurotrophic factor; CREB, cAMP response element-binding protein; GAD, generalized anxiety disorder; HPA, hypothalamic–pituitary–adrenal; Nrf2, nuclear factor erythroid 2-related factor 2; RCT, randomized controlled trial.

These study types should not be interpreted as providing equivalent levels of evidence. Most preclinical studies used chronic or induced depression-related models, whereas two studies relied on acute forced swim or tail suspension assays in non-stressed animals. The chronic or induced models provide a broader experimental context for depression-related phenotypes, while acute behavioral assays primarily indicate short-term antidepressant-like activity and have more limited translational relevance [33,34]. Nevertheless, both approaches remain preclinical and cannot establish clinical efficacy. The single human study was included because, despite enrolling participants with generalized anxiety disorder, it reported a depression-related outcome and therefore met the prespecified eligibility criteria [21]. However, it was small, uncontrolled, open-label, and did not enroll participants with diagnosed major depressive disorder. Its findings therefore provide only limited and indirect clinical evidence regarding depression. No depression-specific clinical trial of *C. asiatica* was identified. Overall, the available evidence supports biological plausibility and preclinical antidepressant-like potential but does not establish the clinical efficacy of *C. asiatica* for depression. The overall evidence map, including the studied preparations, experimental models, depression-linked mechanisms, and major translational gaps, is summarized in Figure 2. These observations are best interpreted alongside more mature clinical evidence for other plant-derived antidepressant interventions, which will be discussed in the following subsection.

### 4.2. Comparison with Other Plant-Derived Antidepressant Evidence

The mechanistic profile reported for *C. asiatica* shares several features with other botanical interventions investigated for depression, including modulation of monoaminergic, neurotrophic, inflammatory, oxidative, and stress-response pathways [7,11,35]. However, mechanistic similarities should not be interpreted as evidence of comparable clinical efficacy. St. John’s wort, saffron, and curcumin have a more mature clinical evidence base, including randomized controlled trials and meta-analyses in depressed populations [36,37,38,39,40]. In contrast, evidence for *C. asiatica* remains predominantly preclinical, with limited human evidence and no depression-specific clinical trial identified. Thus, *C. asiatica* remains at an earlier translational stage than these better-studied botanical interventions. These comparisons are presented only to contextualize the current evidence gap and not as direct comparisons of efficacy.

### 4.3. Translational Gaps and Clinical Implications

The clinical evidence supporting *C. asiatica* for depression remains very limited, resting on a single small, open-label human study that was not directed at major depressive disorder [21]. The current registered clinical pipeline does not yet address this gap. Although five studies were listed as ongoing or active, none evaluated participants with depression or assessed antidepressant efficacy. The closest CNS-related study, NCT05591027, investigated oral *C. asiatica* in individuals with mild cognitive impairment or Alzheimer’s disease and may provide useful evidence regarding safety, tolerability, oxidative stress modulation, and central target engagement. However, its neurocognitive population and non-depression-specific outcomes preclude inference regarding antidepressant efficacy. Ongoing studies of oral asiaticoside and madecassoside in nonpsychiatric populations may provide preparation-specific human exposure and safety data, whereas the oil massage and intracanal studies have limited translational relevance to depression. Consequently, the existing evidence and registered clinical pipeline remain insufficient to support conclusions regarding antidepressant efficacy, and the findings of this review should be regarded as hypothesis-generating rather than confirmatory.

Additional translational gaps concern dosing, intervention characterization, pharmacokinetics, and safety. No validated human dose has been established for any *C. asiatica* extract or isolated triterpenoid for depression, and the 500 mg twice-daily regimen used in the single human study should not be interpreted as a validated depression dose [21]. The included preclinical studies also varied substantially in dose, route, and treatment duration, while several extract-based studies incompletely reported composition or standardization. Safety reporting was limited or absent across the included studies. Although external literature provides some information on the safety and pharmacokinetics of standardized *C. asiatica* preparations [11,41,42,43], these data were not derived from depression-specific trials and cannot be directly extrapolated to the interventions evaluated in this review.

Finally, most reported mechanisms remain associative and rely primarily on concurrent changes in biomarkers. Although a small number of studies used pharmacological antagonists to provide more direct experimental support for specific pathways [28,30], these findings do not establish causal mechanisms across different preparations or experimental models. Taken together, the associative nature of the mechanistic evidence and the limitations in clinical evidence, dosing, standardization, and safety preclude recommending any *C. asiatica* extract or isolated triterpenoid for the treatment of depression at present. These limitations also inform the research priorities discussed below.

### 4.4. Limitations

This review has several limitations. First, this scoping review’s objective was to map the available evidence rather than estimate treatment efficacy. Second, meta-analysis was not performed because substantial heterogeneity in study type, experimental model, intervention, dose, route of administration, treatment duration, outcome measures, and mechanistic endpoints precluded meaningful quantitative pooling. Third, no formal certainty-of-evidence assessment was conducted. Therefore, the conclusions describe the scope and nature of the existing literature and should not be interpreted as proof of clinical effectiveness. Fourth, eligibility was restricted to full-text articles written in English, which may have excluded relevant studies published in other languages. Fifth, unpublished studies, traditional medicine reports written in local languages, theses, and other non-indexed literature may not have been captured, and these omissions may have affected the completeness of the evidence map. Sixth, the modest number of included studies appears to reflect the limited depression-focused literature on *C. asiatica* and the application of predefined eligibility criteria that require depression-related outcomes or mechanisms explicitly linked to depression. In addition, the database search did not include PsycINFO or Embase. Although searching the selected databases and screening the reference lists of included studies provided broad coverage, relevant psychiatric, behavioral, or pharmacological studies indexed exclusively in PsycINFO or Embase may have been missed.

The included studies also had notable limitations. First, the evidence base is dominated by non-human studies, with only one human clinical study, which substantially constrains any inferences regarding clinical relevance. Second, many animal studies relied on single depression-related models or acute behavioral assays, such as forced swim and tail suspension tests, in non-stressed animals, which do not fully represent the chronic and multifactorial nature of clinical depression. Third, the included studies varied widely in terms of *C. asiatica* preparation, isolated compounds, dose, route of administration, treatment duration, comparator, and outcome measures, limiting cross-study comparability. Fourth, several studies incompletely reported extract standardization, triterpenoid composition, or dosing rationale, and many lacked direct mechanistic validation. Fifth, safety reporting was limited or absent, with insufficient primary data on adverse events, toxicity, mortality, tolerability, and laboratory safety. Preclinical sample sizes were also generally small, with reported group sizes ranging from 6 to 12, and the animals were predominantly male. Accordingly, the overall consistency of favorable findings across studies should not be interpreted as evidence of high certainty, particularly because formal risk-of-bias and certainty-of-evidence assessments were not conducted.

Furthermore, the available evidence does not specifically address major depressive disorder, including severe depression, because the only human study involved participants with generalized anxiety disorder and associated depressive symptoms. Evidence regarding use in patients with concomitant diseases or alongside conventional antidepressants is also insufficient. Taken together, these limitations constrain the clinical interpretation of the available evidence and reinforce the need for more rigorous preclinical and clinical research.

### 4.5. Future Perspectives

Future research on *C. asiatica* in the context of depression would benefit from greater standardization and more translationally informative preclinical designs. Because crude botanical extracts may vary considerably in composition and the concentration of a single chemical marker may not fully reflect the overall biological activity, future studies should prioritize standardized preparations characterized by comprehensive chromatographic fingerprinting rather than a single marker compound [44,45,46]. Ideally, standardization should use validated bioactive markers—triterpenoids demonstrated to be extractable, bioavailable, and pharmacologically active—rather than relying solely on general pharmacopeial markers [47]. To this end, studies should report on the plant parts used, extraction solvents, phytochemical profiles, marker compounds, triterpenoid content, batch consistency, and stability. Among the isolated triterpenoids, asiaticoside and asiatic acid have the largest preclinical evidence base and therefore warrant continued investigation, whereas madecassoside and madecassic acid remain comparatively underrepresented. These observations should not be interpreted as evidence that isolated triterpenoids are preferable to standardized *C. asiatica* extracts for further clinical development.

To narrow these translational gaps, future preclinical studies should incorporate dose–response evaluation, pharmacokinetic profiling, and assessment of brain penetration and bioavailability. Human pharmacokinetic studies have characterized the systemic exposure and biotransformation of triterpenoids from standardized *C. asiatica* preparations, including the conversion of the parent glycosides to their corresponding aglycones [42,43]. Interspecies differences and limitations of animal models may further complicate translation of preclinical dosing to humans [33]. In this regard, in vitro blood–brain barrier models indicate that asiaticoside, madecassoside, and asiatic acid possess high apparent permeability, providing a preliminary pharmacokinetic rationale for studying their central nervous system activity with in vivo confirmation [10]. Future studies should include both sexes where possible, given the higher prevalence of depression and documented sex-specific stress responses in females [33,48]. Findings should also be replicated across multiple depression-related models because no single experimental paradigm captures the full spectrum of human depression [49]. Building on the associative nature of current mechanistic data, future investigations should move beyond correlational biomarker tracking toward causal validation using selective receptor antagonists or genetic models [49,50]; notably, one of the included studies employed a TrkB antagonist, providing a precedent for this approach.

Clinical development should proceed cautiously and focus on a clearly defined intervention, whether a standardized *C. asiatica* extract or an isolated triterpenoid. Evidence supporting one intervention type should not be used to infer the safety, pharmacokinetics, dose, or efficacy of another. Phase I studies should establish intervention-specific safety, tolerability, human pharmacokinetics, and dose selection before efficacy is assessed [43]. Subsequent studies should adopt randomized, double-blind, placebo-controlled designs in clearly diagnosed depressive populations. Given the poor reporting quality frequently observed in botanical trials [51], these studies should follow established reporting standards for herbal interventions, including relevant CONSORT extensions or herbal medicine reporting guidance, where applicable [52,53]. Extraction solvents, phytochemical fingerprints, and marker-compound concentrations should be transparently reported. Clinical trials should also specify a priori whether the intervention is administered as monotherapy or as adjunctive therapy alongside conventional antidepressants. This distinction is particularly relevant because one included study evaluated asiatic acid as a potentiator of imipramine and escitalopram [23]. The evidence mapping approach used in the present review may help summarize the relationships among compounds, models, mechanisms, and translational gaps to guide research prioritization. Overall, the current evidence provides a coherent foundation for the biological plausibility and preclinical antidepressant-like activity of *C. asiatica*; however, only rigorous, standardized, and clinically grounded research can determine whether this early promise translates into meaningful benefits for people living with depression.

## 5. Conclusions

This scoping review mapped the available evidence on *C. asiatica* extracts and isolated bioactive triterpenoids in relation to depression-related outcomes and mechanisms. The evidence base was predominantly preclinical, with only one small open-label human study. Whole or standardized *C. asiatica* extracts and isolated triterpenoids should be interpreted as distinct intervention types because their pharmacological properties are not equivalent. Asiaticoside and asiatic acid were the most frequently investigated isolated compounds, while several studies evaluated whole or standardized extracts. Generally favorable antidepressant-like signals were associated with BDNF/CREB-related signaling and neuroplasticity, monoaminergic regulation, attenuation of neuroinflammation and oxidative stress, and HPA axis modulation. Most mechanistic findings were associative, while direct experimental support using pharmacological manipulation was limited to a small number of studies. Accordingly, the reported pathways should not be interpreted as universally established causal mechanisms. The predominance of favorable findings should also not be interpreted as high-certainty evidence, given the methodological limitations of the underlying studies. However, findings from extracts should not be extrapolated to isolated triterpenoids, or vice versa, and the available evidence remains insufficient to establish clinical efficacy or support the therapeutic use of either intervention type for depression. Important gaps remain in intervention characterization, dose selection, pharmacokinetics, safety, validated clinical outcomes, and mechanistic confirmation. Clinical development should therefore proceed cautiously, using a clearly defined and well-characterized intervention, with extracts and isolated compounds evaluated separately. Phase I studies should first examine intervention-specific safety, tolerability, pharmacokinetics, and dose selection. A randomized Phase II proof-of-concept trial should be considered only after these requirements are satisfied. The optimal intervention for further clinical development remains to be determined.

## Figures and Tables

**Table 1:** Intervention details and experimental models.

Author, Year	Country	Study Type	Model/Population	Preparation or Compound	Extract Standardization, if Reported	Dose	Route	Duration	Comparator/Control
Liang, 2008 [26]	China	Animal study	Mice; CMS	Asiaticoside	Not reported	5, 10, 20, and 40 mg/kg	Oral	Acute and 14 days	Vehicle; clomipramine
Jana, 2010 [21]	India	Human clinical study	Patients with generalized anxiety disorder and correlated depression scores (*n* = 33)	*Centella asiatica* hydroethanolic extract	Ursolic acid 1.5–1.8%	500 mg capsule twice daily	Oral	60 days	Not reported
Kalshetty, 2012 [27]	India	Animal study	Rats; OBX	*Centella asiatica* leaf extract (INDCA)	Standardized to asiaticoside 45.74%	3, 10, and 30 mg/kg	Oral	14 days	Sham control; vehicle; imipramine; fluoxetine; desipramine
Luo, 2015 [28]	China	Animal study	Mice; CUMS	Asiaticoside	Purity 98.4%	10, 20, and 40 mg/kg	Oral gavage	4 weeks	Vehicle; fluoxetine
Hou, 2017 [29]	China	Animal study	Rats; CUS	Asiaticoside, with or without borneol	Not reported	300 mg/100 mL, 1 mL/100 g; approximately 30 mg/kg	Oral	Acute and 4 weeks	Vehicle; fluoxetine
Girish, 2020 [30]	India	Animal study	Non-stressed mice; FST/TST assays	Asiatic acid	Not reported	5, 10, and 20 mg/kg	Oral gavage	Acute	Vehicle; fluoxetine
Wang, 2020 [13]	China	Animal study	Mice; CMS	Asiaticoside	Purity >98%	20 and 40 mg/kg	Intragastric	4 weeks	Vehicle; fluoxetine
Jagadeesan, 2022 [31]	Malaysia	Animal study	Rats; CUMS	*Centella asiatica* ethanolic extract	Not reported	200, 400, and 800 mg/kg	Oral	8 weeks	Normal saline; fluoxetine
Bertollo, 2024 [32]	Brazil	Animal study	Rats; MD	*Centella asiatica* hydroalcoholic extract; madecassic acid	Madecassic acid purity >95%	Extract, 30 mg/kg; madecassic acid, 10 mg/kg	Oral gavage	14 days	Vehicle; escitalopram
Mando, 2024 [20]	Malaysia	In vivo/in silico study	Zebrafish; UCS	Asiaticoside, madecassoside, asiatic acid, and madecassic acid	Purity 98%	1.25, 2.5, and 5 mg/kg	Intraperitoneal	Acute	Vehicle; fluoxetine; escitalopram
Ren, 2024 [24]	China	Animal study	Mice; CUMS	Asiaticoside	HPLC purity 95%	10, 20, and 40 mg/kg	Oral gavage	4 weeks	Physiological saline; fluoxetine
Thakurdesai, 2024 [22]	India	Animal study	Rats; CUMS	*Centella asiatica* leaf extract (INDCA-NS)	Standardized to triterpenoids	10, 30, and 100 µg/rat/day	Intranasal	14 days	Vehicle control; buspirone
Poleszak, 2025 [23]	Poland	Animal study	Non-stressed mice; FST assay	Asiatic acid	Not reported	5 mg/kg	Intraperitoneal	Acute	Saline; imipramine; reboxetine; escitalopram
Zhou, 2025 [25]	China	Animal study	Mice; CRS	Asiaticoside	Purity >99%	20 mg/kg	Oral	28 days	Normal vehicle; CRS vehicle

Note. CMS, chronic mild stress; CRS, chronic restraint stress; CUMS, chronic unpredictable mild stress; CUS, chronic unpredictable stress; FST, forced swim test; HPLC, high-performance liquid chromatography; INDCA, standardized *Centella asiatica* leaf extract; INDCA-NS, intranasal standardized *Centella asiatica* leaf extract; MD, maternal deprivation; OBX, olfactory bulbectomy; TST, tail suspension test; UCS, unpredictable chronic stress. “Not reported” indicates that the information is not available in the extracted full-text data. For studies using non-stressed animals, the model/population column refers to antidepressant-like behavioral assays rather than chronic depression models.

**Table 2:** Depression-related outcomes and main findings.

Author, Year	Depression-Related Model/Outcome	Behavioral or Clinical Measure	Main Finding	Direction of Effect
Liang, 2008 [26]	CMS and acute antidepressant-like models in mice	Splash test, coat state, TST, FST	Asiaticoside decreased immobility time in the TST and FST and counteracted CMS-induced deterioration in coat state and grooming behavior.	Improved
Jana, 2010 [21]	Generalized anxiety disorder with correlated depression in humans	BPRS; self-reported depression scale	*C. asiatica* extract reduced the depression index by 10.2% at 30 days and by 21.8% at 60 days.	Improved
Kalshetty, 2012 [27]	OBX model in rats	OFT, EPM	Standardized *C. asiatica* extract normalized OBX-induced hyperactivity, hyperemotionality, and anxiety-related behavior.	Improved
Luo, 2015 [28]	CUMS in mice	SPT, FST	Asiaticoside significantly reversed CUMS-induced reduction in sucrose preference and decreased immobility time in the FST.	Improved
Hou, 2017 [29]	CUS in rats	Modified FST, NSFT, sucrose preference	Asiaticoside alone showed limited overt antidepressant-like effects; however, asiaticoside combined with borneol reduced immobility time, increased sucrose intake, and increased BDNF and 5-HT levels.	Mixed
Girish, 2020 [30]	Acute antidepressant-like behavioral assays in non-stressed mice	FST, TST	Asiatic acid reduced immobility time in the TST and FST. Pharmacological antagonist experiments suggested involvement of the serotonergic and noradrenergic systems.	Improved
Wang, 2020 [13]	CMS in mice	SPT, TST, FST	Asiaticoside reversed reduced sucrose consumption and decreased immobility time in the TST and FST.	Improved
Jagadeesan, 2022 [31]	CUMS in rats	Hippocampal ultrastructure; oxidative stress biomarkers	*C. asiatica* extract prevented CUMS-induced hippocampal ultrastructural abnormalities and improved oxidative stress markers by reducing MDA and increasing SOD.	Improved
Bertollo, 2024 [32]	MD in rats	FST, OFT	*C. asiatica* extract and madecassic acid reduced immobility time in the FST, reversing MD-induced depressive-like behavior.	Improved
Mando, 2024 [20]	UCS in zebrafish	OFT; whole-body cortisol	Madecassoside, asiaticoside, and asiatic acid counteracted stress-induced locomotor retardation. Madecassoside and asiaticoside reduced elevated cortisol levels in stressed zebrafish.	Improved
Ren, 2024 [24]	CUMS in mice	SPT, FST, OFT	Asiaticoside increased sucrose preference and reduced immobility time in the FST, with additional improvement in OFT-related behavioral outcomes.	Improved
Thakurdesai, 2024 [22]	CUMS in rats	SPT, MBT, RIT	INDCA-NS significantly prevented CUMS-induced reduction in sucrose consumption, with dose-dependent significance reported across groups. It also reduced CUMS-induced anxiety- and aggression-related behaviors.	Improved
Poleszak, 2025 [23]	Acute antidepressant-like behavioral assays in non-stressed mice	FST, TST	A sub-active dose of asiatic acid potentiated the antidepressant-like effects of imipramine, reboxetine, and escitalopram, reducing immobility time.	Improved
Zhou, 2025 [25]	CRS in mice	SPT, FST	Asiaticoside increased sucrose preference and decreased immobility time in CRS-exposed mice.	Improved

Note. 5-HT, 5-hydroxytryptamine/serotonin; BDNF, brain-derived neurotrophic factor; BPRS, Brief Psychiatric Rating Scale; CMS, chronic mild stress; CRS, chronic restraint stress; CUMS, chronic unpredictable mild stress; CUS, chronic unpredictable stress; EPM, elevated plus maze; FST, forced swim test; INDCA-NS, intranasal standardized *Centella asiatica* leaf extract; MBT, marble burying test; MD, maternal deprivation; MDA, malondialdehyde; NSFT, novelty-suppressed feeding test; OBX, olfactory bulbectomy; OFT, open field test; RIT, resident intruder test; SOD, superoxide dismutase; SPT, sucrose preference test; TST, tail suspension test; UCS, unpredictable chronic stress. “Direction of effect” is a descriptive summary of the overall depression-related or antidepressant-like outcome reported by each study and does not represent effect size, study quality, reproducibility, or certainty of evidence. For studies using non-stressed animals, the listed outcomes refer to acute antidepressant-like behavioral assays rather than chronic depression models.

**Table 3:** Depression-linked mechanisms, safety, and translational gaps.

Author, Year	Mechanistic Pathway Assessed	Specific Markers	Main Mechanistic Findings	Safety Findings	Translational Gap
Liang, 2008 [26]	Behavioral antidepressant-like effects; no biochemical mechanism assessed	Not reported	Asiaticoside produced antidepressant-like behavioral effects, but biochemical mechanisms were not evaluated.	Not reported	Mechanism not validated; no confirmation in humans; limited safety reporting
Jana, 2010 [21]	Clinical symptom outcomes; no specific mechanistic pathway assessed	BPRS; self-reported anxiety and depression scales	*C. asiatica* extract reduced anxiety, stress, and correlated depression scores in human participants.	Not reported	Small clinical sample; single-arm design; short treatment duration; limited safety reporting
Kalshetty, 2012 [27]	Behavioral and physiological outcomes in OBX model	Not reported	Standardized *C. asiatica* extract reversed behavioral and physiological changes in the OBX model, but specific biochemical markers were not measured.	Not reported	Mechanism not validated; no confirmation in humans; limited safety reporting
Luo, 2015 [28]	BDNF signaling; synaptic plasticity	BDNF; synapsin I; PSD-95; TrkB antagonist K252a	Asiaticoside upregulated hippocampal BDNF, PSD-95, and synapsin I; effects were abolished by the TrkB antagonist K252a.	Not reported	No confirmation in humans; single animal model; limited safety reporting
Hou, 2017 [29]	Monoaminergic system; BDNF signaling; neuroinflammation	5-HT; NE; BDNF; TNF-α	Asiaticoside combined with borneol increased hippocampal 5-HT and BDNF and reduced TNF-α levels.	Not reported	No confirmation in humans; single animal model; limited safety reporting
Girish, 2020 [30]	Monoaminergic system	WAY-100635; ketanserin; ondansetron; prazosin; yohimbine	Antidepressant-like effects of asiatic acid were blocked by serotonergic and noradrenergic receptor antagonists, suggesting involvement of these monoaminergic systems.	Not reported	Mechanism not clinically validated; no confirmation in humans; limited safety reporting
Wang, 2020 [13]	Monoaminergic system; neuroinflammation; PKA/pCREB/BDNF signaling	5-HT; NE; IL-1β; IL-6; TNF-α; pNF-κB p65; NLRP3; caspase-1; cAMP; PKA; pVASP; pCREB; BDNF	Asiaticoside increased 5-HT, NE, cAMP, PKA, pVASP, pCREB, and BDNF while reducing inflammatory cytokines and NLRP3 inflammasome-related markers.	Not reported	No confirmation in humans; single animal model; limited safety reporting
Jagadeesan, 2022 [31]	Oxidative stress; mitochondrial and ultrastructural integrity; synaptic plasticity	MDA; SOD; hippocampal ultrastructure of mitochondria, nucleus, myelin sheath, and synapses	*C. asiatica* extract attenuated CUMS-induced hippocampal ultrastructural abnormalities, decreased MDA, and increased SOD activity.	Not reported	Extract composition unclear; no confirmation in humans; limited safety reporting
Bertollo, 2024 [32]	Neuroinflammation; oxidative stress	IL-1β; IL-6; MPO; TBARS; PSH; NPSH	*C. asiatica* extract and madecassic acid reduced hippocampal IL-1β and IL-6 and improved oxidative stress-related markers in serum and hippocampus.	Not reported	Extract composition partly unclear; no confirmation in humans; limited safety reporting
Mando, 2024 [20]	HPA axis; monoaminergic system; in silico transporter binding	Whole-body cortisol; SERT docking	Asiaticoside and madecassoside reduced elevated cortisol in stressed zebrafish; molecular docking suggested binding affinity to SERT central and allosteric sites.	Not reported	No confirmation in humans; in silico mechanism requires experimental validation; limited safety reporting
Ren, 2024 [24]	Gut microbiota; monoaminergic system; BDNF signaling; neuroinflammation; HPA axis	*Alistipes*; *Desulfovibrio*; *Lachnospiraceae*; *Lactobacillus*; Firmicutes; SCFAs; 5-HT1A; BDNF; 5-HT; IL-6; TNF-α; IL-10; CRH; CORT	Asiaticoside modulated gut microbiota and SCFAs, increased hippocampal 5-HT1A, BDNF, and serum 5-HT, and reduced inflammatory and HPA-axis markers.	Not reported	No confirmation in humans; single animal model; limited safety reporting
Thakurdesai, 2024 [22]	HPA axis; BDNF signaling	Cortisol; BDNF	INDCA-NS prevented stress-induced elevation of hypothalamic cortisol but did not significantly affect BDNF levels.	Not reported	No confirmation in humans; single animal model; limited safety reporting
Poleszak, 2025 [23]	Oxidative stress	Catalase; GPx; TBARS	Asiatic acid combined with antidepressants increased antioxidant enzyme activity and reduced lipid peroxidation markers in the brain.	Not reported	No confirmation in humans; acute model only; limited safety reporting
Zhou, 2025 [25]	BDNF/TrkB signaling; oxidative stress/Nrf2; ferroptosis; neuroinflammation; mitochondrial and synaptic integrity	BDNF; pTrkB; PSD-95; synaptophysin; DCX; Iba1; pNrf2; GPX4; SLC7A11; HO-1; FLC; transferrin receptor	Asiaticoside activated BDNF/TrkB and Nrf2/GPX4/SLC7A11 signaling, improved synaptic and mitochondrial integrity, inhibited microglial activation, and reduced ferroptosis-related changes.	Not reported	No confirmation in humans; single animal model; limited safety reporting

Note. 5-HT, 5-hydroxytryptamine/serotonin; 5-HT1A, serotonin 1A receptor; BDNF, brain-derived neurotrophic factor; BPRS, Brief Psychiatric Rating Scale; cAMP, cyclic adenosine monophosphate; CORT, corticosterone; CRH, corticotropin-releasing hormone; CUMS, chronic unpredictable mild stress; DCX, doublecortin; FLC, ferritin light chain; GPx, glutathione peroxidase; GPX4, glutathione peroxidase 4; HO-1, heme oxygenase-1; HPA, hypothalamic–pituitary–adrenal; Iba1, ionized calcium-binding adaptor molecule 1; IL, interleukin; INDCA-NS, intranasal standardized *Centella asiatica* leaf extract; MDA, malondialdehyde; MPO, myeloperoxidase; NE, norepinephrine; NF-κB, nuclear factor kappa B; NLRP3, nucleotide-binding oligomerization domain-like receptor family pyrin domain containing 3; NPSH, non-protein sulfhydryl; Nrf2, nuclear factor erythroid 2-related factor 2; OBX, olfactory bulbectomy; pCREB, phosphorylated cAMP response element-binding protein; PKA, protein kinase A; PSD-95, postsynaptic density protein 95; pTrkB, phosphorylated tropomyosin receptor kinase B; pVASP, phosphorylated vasodilator-stimulated phosphoprotein; SCFA, short-chain fatty acid; SERT, serotonin transporter; SLC7A11, solute carrier family 7 member 11; SOD, superoxide dismutase; TBARS, thiobarbituric acid-reactive substances; TNF-α, tumor necrosis factor-alpha; TrkB, tropomyosin receptor kinase B. “Not reported” indicates that safety, toxicity, adverse effects, mortality, or tolerability outcomes are not available in the extracted full-text data. Translational gaps are interpreted from the study design, model, intervention reporting, outcome reporting, and extent of mechanistic validation.

## Data Availability

All data generated or analyzed during this study are included in this article and its Supplementary Materials.

## Supplementary Materials

The following supporting information can be downloaded at https://www.mdpi.com/article/10.3390/life16081345/s1, Table S1: Complete search strategies and number of records retrieved from each source; Supplementary File S1: PRISMA-ScR checklist.
