# Supplementary material for: Depression-Related Mechanistic and Translational Evidence for Centella asiatica and Its Triterpenoids: A Scoping Review
Source: Life (Basel). 2026 Aug 16;16(8):1345. doi: 10.3390/life16081345 (PMC13514948; doi:10.3390/life16081345)
Supplement: Supplementary file 1 [file life-16-01345-s001.zip › Table S1. Complete Search Strategies.pdf]

Supplementary Table S1. Complete search strategies and number of records retrieved from each source

Database	Search strings	Records
Scopus	TITLE-ABS-KEY (("Centella asiatica" OR "C. asiatica" OR "C asiatica" OR "Gotu kola" OR "Hydrocotyle asiatica" OR "Indian pennywort" OR (centella W/3 extract*) OR (centella W/3 triterpene*) OR (centella W/3 triterpenoid*) OR ("gotu kola" W/3 extract*) OR ("gotu kola" W/3 triterpene*) OR ("gotu kola" W/3 triterpenoid*) OR "asiatic acid" OR asiaticoside OR "madecassic acid" OR madecassoside OR "madasiatic acid" OR TECA OR TTFCA OR "total triterpenes of Centella asiatica" OR "total triterpenic fraction of Centella asiatica") AND (depress* OR "depressive-like" OR "depression-like" OR antidepress* OR "antidepressant-like" OR "major depressive disorder" OR MDD OR mood OR anxi* OR stress OR "stress-related" OR "chronic stress" OR "chronic unpredictable mild stress" OR "chronic mild stress" OR CUMS OR "forced swim" OR "tail suspension" OR "sucrose preference" OR "HPA axis" OR "hypothalamic-pituitary-adrenal" OR cortisol OR corticosterone OR monoamine* OR serotonin OR dopamine OR norepinephrine OR noradrenaline OR BDNF OR CREB OR neuroinflamm* OR neuropsychiatr* OR neurobehavior* OR "oxidative stress" OR Nrf2 OR mitochondri* OR neuroplastic* OR neurogenesis OR synapt*) AND (brain OR cerebral OR hippocamp* OR cortex OR cortical OR neuron* OR neuronal OR astrocyt* OR microglia* OR neuro* OR CNS OR "central nervous system" OR psychiatric OR psychological OR behavioral OR behavioural OR emotion* OR cognition)) AND PUBYEAR < 2027 AND (LIMIT-TO (LANGUAGE , "English"))	683
PubMed/MEDLINE	("Centella asiatica"[Title/Abstract] OR "C. asiatica"[Title/Abstract] OR "C asiatica"[Title/Abstract] OR "Gotu kola"[Title/Abstract] OR "Hydrocotyle asiatica"[Title/Abstract] OR "Indian pennywort"[Title/Abstract] OR "Centella"[Title/Abstract] OR "asiatic acid"[Title/Abstract] OR asiaticoside[Title/Abstract] OR "madecassic acid"[Title/Abstract] OR madecassoside[Title/Abstract] OR "madasiatic acid"[Title/Abstract] OR TECA[Title/Abstract] OR TTFCA[Title/Abstract] OR "total triterpenes of Centella asiatica"[Title/Abstract] OR "total triterpenic fraction of Centella asiatica"[Title/Abstract]) AND (depress*[Title/Abstract] OR "depressive-like"[Title/Abstract] OR "depression-like"[Title/Abstract] OR antidepress*[Title/Abstract] OR "antidepressant-like"[Title/Abstract] OR "major depressive disorder"[Title/Abstract] OR MDD[Title/Abstract] OR mood[Title/Abstract] OR anxi*[Title/Abstract] OR	47

	stress[Title/Abstract] OR "stress-related"[Title/Abstract] OR "chronic stress"[Title/Abstract] OR "chronic unpredictable mild stress"[Title/Abstract] OR "chronic mild stress"[Title/Abstract] OR CUMS[Title/Abstract] OR "forced swim"[Title/Abstract] OR "tail suspension"[Title/Abstract] OR "sucrose preference"[Title/Abstract] OR "HPA axis"[Title/Abstract] OR "hypothalamic-pituitary-adrenal"[Title/Abstract] OR cortisol[Title/Abstract] OR corticosterone[Title/Abstract] OR monoamine*[Title/Abstract] OR serotonin[Title/Abstract] OR dopamine[Title/Abstract] OR norepinephrine[Title/Abstract] OR noradrenaline[Title/Abstract] OR BDNF[Title/Abstract] OR CREB[Title/Abstract] OR neuroinflamm*[Title/Abstract] OR neuropsychiatr*[Title/Abstract] OR neurobehavior*[Title/Abstract] OR neurobehaviour*[Title/Abstract] OR "oxidative stress"[Title/Abstract] OR Nrf2[Title/Abstract] OR mitochondri*[Title/Abstract] OR neuroplastic*[Title/Abstract] OR neurogenesis[Title/Abstract] OR synapt*[Title/Abstract]) AND (brain[Title/Abstract] OR cerebral[Title/Abstract] OR hippocamp*[Title/Abstract] OR cortex[Title/Abstract] OR cortical[Title/Abstract] OR neuron*[Title/Abstract] OR neuronal[Title/Abstract] OR astrocyt*[Title/Abstract] OR microglia*[Title/Abstract] OR neuro*[Title/Abstract] OR CNS[Title/Abstract] OR "central nervous system"[Title/Abstract] OR psychiatric[Title/Abstract] OR psychological[Title/Abstract] OR behavioral[Title/Abstract] OR behavioural[Title/Abstract] OR emotion*[Title/Abstract] OR cognition[Title/Abstract]) AND english[Language]	
Cochrane Library/CENTRAL Trials	(("Centella asiatica" OR "C asiatica" OR "C. asiatica" OR "Gotu kola" OR "Hydrocotyle asiatica" OR "Indian pennywort" OR Centella OR "asiatic acid" OR asiaticoside OR "madecassic acid" OR madecassoside OR "madasiatic acid" OR TECA OR TTFCA OR "total triterpenes of Centella asiatica" OR "total triterpenic fraction of Centella asiatica"):ti,ab,kw)<br>AND ((depress* OR "depressive like" OR "depression like" OR antidepress* OR "antidepressant like" OR "major depressive disorder" OR MDD OR mood OR anxi* OR stress OR "stress related" OR "chronic stress" OR "chronic unpredictable mild stress" OR "chronic mild stress" OR CUMS OR "forced swim" OR "tail suspension" OR "sucrose preference" OR "HPA axis" OR "hypothalamic pituitary adrenal" OR cortisol OR corticosterone OR monoamine* OR serotonin OR dopamine OR norepinephrine OR noradrenaline OR BDNF OR CREB OR neuroinflamm* OR neuropsychiatr* OR neurobehavior* OR neurobehaviour* OR "oxidative stress" OR "NF kB" OR "NF-kB" OR NLRP3 OR Nrf2 OR mitochondri* OR neuroplastic* OR neurogenesis OR synapt*):ti,ab,kw)	24

	AND ((brain OR cerebral OR hippocamp* OR cortex OR cortical OR neuron* OR neuronal OR astrocyt* OR microglia* OR neuro* OR CNS OR "central nervous system" OR psychiatric OR psychological OR behavioral OR behavioural OR emotion* OR cognition):ti,ab,kw)	
ClinicalTrials.gov	("Centella asiatica" OR "C asiatica" OR "C. asiatica" OR "Gotu kola" OR "Hydrocotyle asiatica" OR "Indian pennywort" OR Centella OR "asiatic acid" OR asiaticoside OR "madecassic acid" OR madecassoside OR "madasiatic acid" OR TECA OR TTFCA OR "total triterpenes of Centella asiatica" OR "total triterpenic fraction of Centella asiatica") AND (depression OR depressive OR "depressive symptoms" OR "major depressive disorder" OR MDD OR mood OR anxiety OR anxious OR stress OR "psychological stress" OR "mental health" OR wellbeing OR "well-being")	27

Note. CENTRAL, Cochrane Central Register of Controlled Trials; MEDLINE, Medical Literature Analysis and Retrieval System Online.
